# Reduced expression of argininosuccinate synthetase 1 has a negative prognostic impact in patients with pancreatic ductal adenocarcinoma

**DOI:** 10.1371/journal.pone.0171985

**Published:** 2017-02-10

**Authors:** Qingqing Liu, John Stewart, Hua Wang, Asif Rashid, Jun Zhao, Matthew H. Katz, Jeffrey E. Lee, Jason B. Fleming, Anirban Maitra, Robert A. Wolff, Gauri R. Varadhachary, Sunil Krishnan, Huamin Wang

**Affiliations:** 1 Department of Pathology, the University of Texas MD Anderson Cancer Center, Houston, Texas, United States of America; 2 Department of Gastrointestinal Medical Oncology, the University of Texas MD Anderson Cancer Center, Houston, Texas, United States of America; 3 Department of Surgical Oncology, the University of Texas MD Anderson Cancer Center, Houston, Texas, United States of America; 4 Department of Radiation Oncology, the University of Texas MD Anderson Cancer Center, Houston, Texas, United States of America; University of Nebraska Medical Center, UNITED STATES

## Abstract

Argininosuccinate synthetase 1 (ASS1), the rate-limiting enzyme for arginine biosynthesis, is expressed in many types of human malignancies. Recent studies showed that ASS1 may have tumor suppressor function and that ASS1 deficiency is associated with clinical aggressiveness in nasopharyngeal carcinoma, myxofibrosarcomas and bladder cancer. The goal of this study was to evaluate the prognostic impact of ASS1 expression in patients with pancreatic ductal adenocarcinoma (PDAC). Our study included two independent cohorts: untreated cohort, which was comprised of 135 patients with PDAC who underwent pancreatoduodenectomy (PD) without pre-operative neoadjuvant therapy, and treated cohort, which was comprised of 122 patients with PDAC who have completed neoadjuvant therapy and PD. The expression level of ASS1 was evaluated by immunohistochemistry and the results were correlated with clinicopathologic parameters and survival using SPSS statistics. Our study showed that 12% of PDAC in untreated cohort and 15% of PDAC in treated cohort has low expression of ASS1 (ASS1-low). ASS1-low was associated with higher recurrence (*p* = 0.045), shorter disease-free survival (DFS, 4.8 ± 1.6 months vs 15.3 ± 2.2 months, *p* = 0.001) and shorter overall survival (OS, 14.6 ± 6.4 months vs 26.5 ± 3.5 months, *p* = 0.005) in untreated cohort and shorter OS in treated cohort compared to ASS1-high tumors. In multivariate analysis, ASS1-low (HR: 0.45, 95% CI: 0.26–0.79, *p* = 0.005) was an independent prognostic factor for DFS in untreated cohort and an independent prognostic factor for OS (HR: 0.56, 95% CI: 0.32–0.97, *p* = 0.04) in treated cohort. Our results provide supporting evidence for future clinical trial using arginine deprivation agents either alone or in combination with conventional chemotherapy in treating pancreatic cancer.

## Introduction

Pancreatic ductal adenocarcinoma (PDAC), the most common histological subtype of pancreatic cancer, is a highly aggressive disease with a 5-yr survival rate of 6% [1]. Surgical resection is the only possible way of curing PDAC. However, less than 20% of patients have resectable disease at the time of diagnosis [2]. Patients with surgically unresectable PDAC at diagnosis are often due to local invasion and/or distant metastasis and have a median survival of less than 6 months. Systemic chemotherapies or chemoradiation therapies remains the standard treatment approach for these patients. However, the benefits of these therapies are limited because PDAC is frequently resistant or develop resistance quickly to conventional therapeutic agents. Recent studies have been focused on identification of more effective therapeutic agents that target the tumor at the molecular level or modulate host immune response, the so-called cancer immunotherapy [3], or agents that can interfere with tumor metabolism [4].

Arginine deprivation therapy combined with the standard chemotherapy has been proposed to treat PDAC since some PDAC are argininosuccinate synthetase 1 (ASS1) deficient and their growth is arginine-dependent (a phenomenon described as “auxotrophy”) [5, 6]. In the susceptible tumors, ASS1 is not up-regulated even when arginine is deprived [7, 8]. In contrast, arginine is produced in normal human cells from citrulline via the urea cycle by the catalytic actions of ASS and argininosuccinate lyase, making it a non-essential amino acid [9]. Systemic deprivation of arginine can be used alone or in combination with other chemotherapy agents to induce tumor cell death in arginine-dependent PDAC. Recently two groups almost simultaneously reported that a novel anticancer enzyme degrading intracellular arginine, pegylated arginine deiminase (PEG-ADI), synergistically augments the cytotoxicity of gemcitabine on PDAC through induction of metabolic stress or inhibition of NF-κB signaling [5, 10]. Arginine deprivation thus may offer a therapeutic opportunity for PDAC deficient in arginine synthesis.

Interestingly, besides it role in arginine synthesis, *Ass1* gene is recently identified as a novel tumor suppressor gene in myxofibrosarcomas [11]. ASS1 has been shown to play an important role in inhibition of tumor cell proliferation via induction of G1 arrest, as well as inhibition of tumor cell migration, invasion and tumor angiogenesis. Loss of ASS1 expression is associated with clinical aggressiveness of the disease [11]. Similar observations have been reported in bladder cancer [12] and nasopharyngeal carcinoma [13]. The prognostic significance of ASS1 in PDAC has not been reported. We therefore in this study examined if ASS1 expression has a prognostic impact in PDAC treated with PD, with or without neoadjuvant therapy. We also examined if neoadjuvant therapy affects ASS1 expression in these patients. Our study will help identify important prognostic factors affecting the survival in patients with PDAC, as well as patients who might benefit from arginine deprivation therapy.

## Materials and methods

### Study populations, patient characteristic, treatment sequencing and follow-up

This study was approved by the Institutional Review Board of the University of Texas M. D. Anderson Cancer Center, Houston, TX. Cases were retrieved from the surgical pathology files of the Department of Pathology, University of Texas M. D. Anderson Cancer Center. Our study population consisted of two independent cohorts: (1) Untreated cohort, which was comprised of 135 patients with stage II PDAC treated with upfront pancreaticoduodenectomy (PD) without pre-operative neoadjuvant therapy (58 women and 77 men with median age of 64.3 years). One patient with stage I disease and four patients with stage IV disease was excluded since the number of cases with either stage I or IV disease was too small to be representative. (2) Treated cohort, which was comprised of 122 patients with PDAC who have completed neoadjuvant therapy before PD at our institution (49 women and 73 men with median age of 62.7 years). Within the treated cohort, 18 patients (14.7%) received fluoropyrimidine-based chemoradiation, 39 patients (32.0%) received gemcitabine-based chemoradiation, 45 patients (36.9%) received gemcitabine followed by gemcitabine-based chemoradiation, 15 patients (12.3%) received gemcitabine followed by fluoropyrimidine -based chemoradiation, and the remaining 5 patients (4.1%) received neoadjuvant systemic chemotherapy alone. All patients in the treated cohort underwent restaging evaluation after completion of neoadjuvant therapy. Only patients, who had no disease progression or metastasis and had no contraindications to major abdominal surgery, were selected for PD.

All cases had confirmed diagnosis of PDAC by histology and were evaluated for clinical presentation, tumor size, differentiation, margins status, extrapancreatic tissue involvement, pathological stage, treatment and clinical outcome based on a standardized system established at our institute. Pathologic stages were grouped according to the AJCC staging manual, 7^th^ edition [14]. Clinical follow-up information was extracted from a prospectively maintained database at the Department of Surgical Oncology at MD Anderson Cancer Center and subsequently verified by reviewing patient medical records and/or the US Social Security Death Index. Recurrence status was updated at each follow-up clinic visit.

### Immunohistochemistry and grading for ASS1 expression

**Fig 1:**
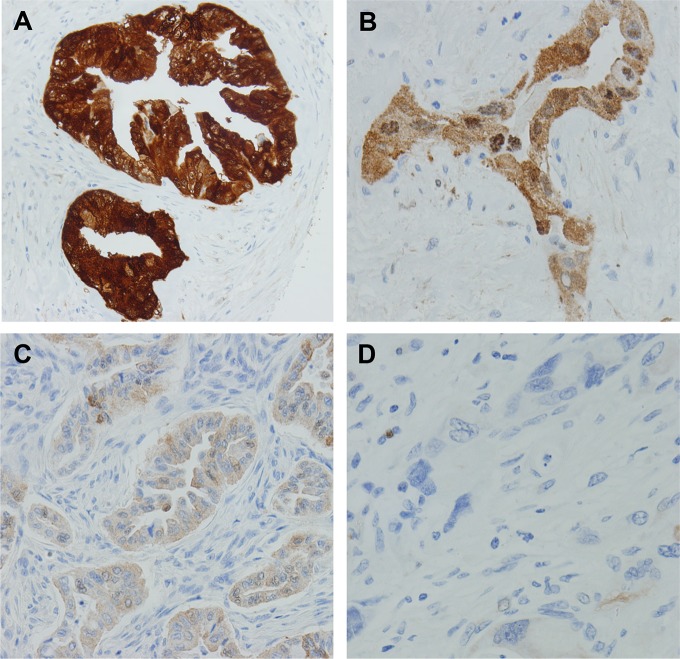
Representative micrographs showing the immunohistochemical (IHC) scores of ASS1 expression (on a scale of 0–3) in pancreatic ductal adenocarcinomas. (A) IHC score 3, (B) IHC score 2, (C) IHC score 1 and (D) IHC score 0.

Immunohistochemical staining for ASS1 was performed on tissue microarray slides, which contain three representative 1.0 mm cores from each patient (two from tumor and one from benign pancreatic tissue). The slides were incubated with mouse anti-ASS1 antibody (1:400, Polaris Group, San Diego, CA). The staining intensity of ASS1 was quantified by visual scoring of staining. The staining results are graded by combined score of the intensity of cytoplasmic staining (0-negative, 1-weak, 2-moderate, and 3-strong, Fig 1) and the percentage of positive tumor cells. The formula for staining score was used: S = p1 x1 + p2 x 2 + p3 x 3 in which p1, p2 and p3 represented fractions of tumor cells representing each staining categories of 1, 2 and 3 respectively. The expression of ASS1 was categorized as ASS1-low and ASS1-high using the combined score 1.5 as a cutoff. The cutoff score of 1.5 was determined based on the previous operational observation that only tumors with an ASS1 immunohistochemical score of 1.5 or lower will respond to pegylated arginine deiminase (PEG-ADI) using the same immunohistochemical protocol and also as reported by other groups previously [15].

### Statistics

The expression of ASS1 was correlated with clinicopathologic parameters and survival using Statistical Package for Social Sciences software (SPSS Inc. Chicago, IL). Categorical variables were compared using the Χ^2^ analysis, Fischer’ exact test or Likelihood ratio. The student’s t test was used to compare the expression of ASS1 in treated cohort to that in untreated cohort. Survival analyses were performed using the Kaplan-Meier method and the statistical significance of difference in survival was evaluated using the log-rank test. Disease-free survival (DFS) was calculated as the time from the date of surgery to the date of first recurrence after surgery in patients with recurrence or to the date of last follow-up in patients without recurrence. Overall survival (OS) was calculated as the time from the date of diagnosis to the date of death or the date of last follow-up if death did not occur. Univariate Cox regression analysis was used to determine the prognostic significance of ASS1 expression and other clinicopathologic characteristics. Cox proportional hazards models were fitted for multivariate analysis. After interactions between the variables were examined, a backward stepwise procedure was used to derive the best-fitting model. All tests are two-sided. P values less than 0.05 are considered statistically significant.

## Results

### ASS1 expression in benign pancreatic tissue, treated and untreated pancreatic cancer samples

**Fig 2:**
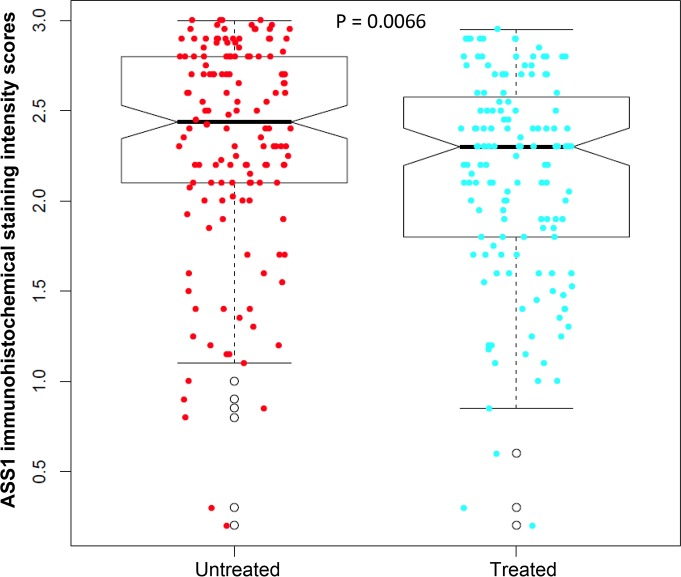
The average immunohistochemical scores of ASS1 expression is significantly lower in treated cohort than that in untreated cohort (*p* = 0.007).

Representative micrographs showing different levels of ASS1 expression among the examined cases are shown in Fig 1. Immunohistochemical stain for ASS1 showed cytoplasmic staining in PDAC cells in cases that were positive for ASS1. The average score of ASS1 expression was 2.13 in treated cohort compared to 2.32 in untreated cohort (*p* = 0.007, Fig 2). There was either no or very low ASS1 expression in cancer associated fibroblasts in all cases.

**Fig 3:**
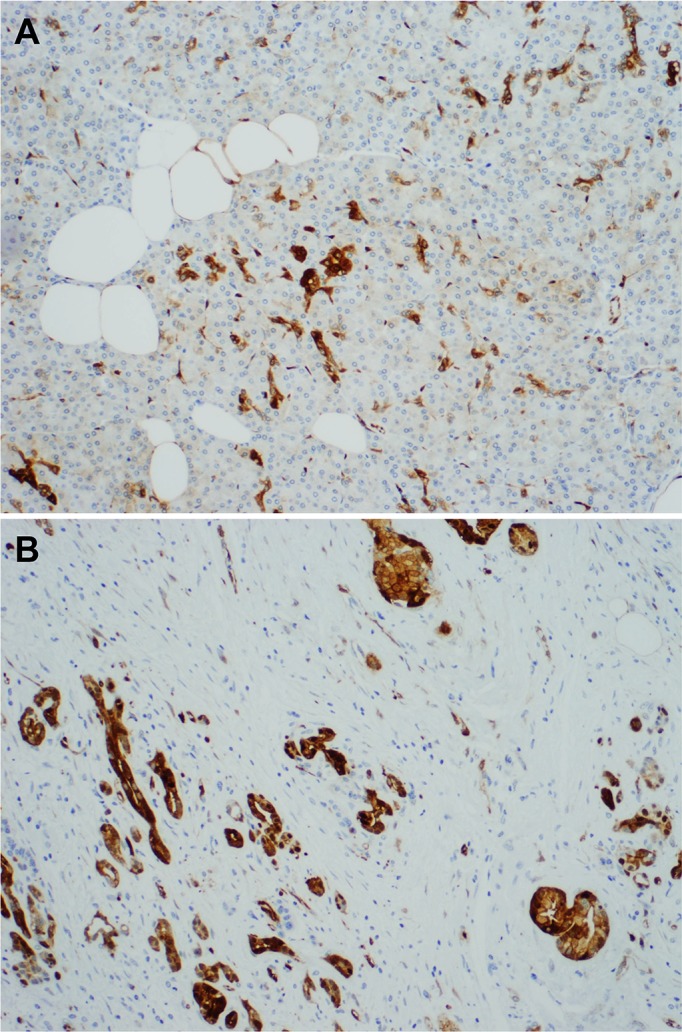
**Representative micrographs showing ASS1 expression in normal pancreas (A) and chronic pancreatitis tissue (B)**.

Among the 163 matched benign pancreatic tissue samples that were available for examination, 124 were histologically normal pancreas and 39 were chronic pancreatitis. In benign pancreas tissue, ASS1expression was detected mainly in pancreatic ductal cells, but not pancreatic islet cells. Pancreatic acinar cell showed negative, weak or moderate staining for ASS1, which may represent non-specific crossing reactions. The average scores of ASS1 expression were 2.31 in normal pancreatic ductal cells and 2.46 in the proliferating pancreatic ductules of chronic pancreatitis respectively. Representative micrographs showing the ASS1 expression in normal pancreas and chronic pancreatitis tissue are shown in Fig 3.

### Correlation of ASS1 expression with clinicopathologic parameters in untreated and treated cohorts

The correlations of ASS1 expression with clinicopathologic characteristics in the untreated and treated cohorts are summarized in Table 1. In the untreated cohort, ASS1 expression correlated significantly with tumor size and tumor recurrence after surgery (Likelihood ratio, p<0.05). All (100%) ASS1-low tumors were greater than 2.0 cm compared to 84% among the ASS1-high tumors (Likelihood ratio, p = 0.02). Local and distant recurrences were present in 37.5% (6/16) and 56.3% (9/16) respectively in ASS1-low tumors compared to 16.0 (19/119) local recurrence and 57.1% (68/119) distant recurrence respectively in ASS1-high tumors (Likelihood ratio, p = 0.045). These correlations, however, were not observed in treated cohort. There were no significant correlations between ASS1 expression and other clinicopathologic parameters including gender, age, tumor differentiation, AJCC stage, lymph node status or margin status in either the untreated or treated cohort (*p* > 0.05).

**Table 1:** Correlation of ASS1 Expression and Clinicopathologic Parameters in Untreated Group and Treated Groups.

	Untreated			Treated		
Characteristics	ASS1-low (%) (n = 16)	ASS1-high (%) (n = 119)	*p* value	ASS1-low (%) (n = 18)*	ASS1-high (%) (n = 104)*	*p* value
**Gender**			0.64			0.91
Female	6 (37.5)	52 (43.7)		7 (38.9)	42 (40.4)	
Male	10 (62.5)	67 (56.3)		11 (61.1)	62 (59.6)	
**Age**			0.55			0.42
<60	6 (37.5)	33 (27.7)		9 (50)	41 (39.4)	
60–70	7 (43.8)	49 (41.2)		7 (38.9)	37 (35.6)	
>70	3 (18.7)	37 (31.1)		2 (11.1)	26 (25)	
**Tumor differentiation**			0.07			0.06
Well-moderate	8 (50)	86 (72.3)		8 (44.4)	70 (67.3)	
Poor	8 (50)	33 (27.3)		10 (55.6)	34 (32.7)	
**Tumor size**			**0.02**			0.8
≤2.0 cm	0 (0)	19 (16.0)		4 (22.2)	26 (25.0)	
>2.0 cm	16 (100)	100 (84.0)		14 (77.8)	78 (75.0)	
**AJCC stage**			0.24			0.84
Stage IIA	2 (12.5)	31 (26.1)		6 (33.3)	33 (31.7)	
Stage IIB	14 (87.5)	88 (73.9)		12 (66.7)	69 (66.3)	
**Lymph node status**			0.24			0.98
Negative	2 (12.5)	31 (26.1)		6 (33.3)	35 (33.7)	
Positive	14 (87.5)	88 (73.9)		12 (66.7)	69 (66.3)	
**Recurrence**			**0.045**			0.44
No recurrence	1 (6.3)	32 (26.9)		3 (17.7)	27 (26.2)	
Local recurrence	6 (37.5)	19 (16.0)		5 (29.4)	23 (22.3)	
Distant recurrence	9 (56.2)	68 (57.1)		9 (52.9)	53 (51.5)	
**Margin status**			0.78			0.06
Negative	14 (87.5)	101 (84.9)		14 (77.8)	96 (92.3)	
Positive	2 (12.5)	18 (15.1)		4 (22.2)	8 (7.7)	

* Due to unavailability of recurrence data, one patient is taken out from ASS1-low and ASS-high groups, respectively.

### ASS1 expression correlated with disease-free and overall survival in untreated cohort

**Fig 4:**
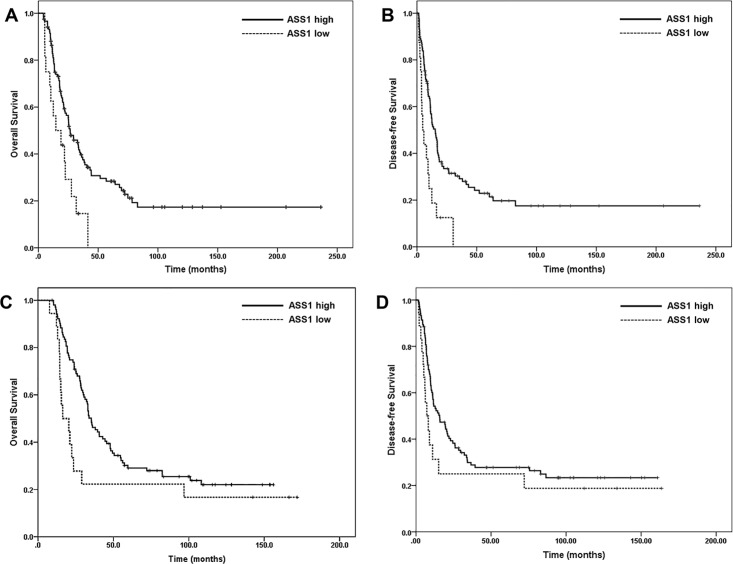
**Kaplan–Meier survival curves showing that low ASS1 expression (ASS1-low) is associated reduced overall survival (*p* = 0.005, A) and disease-free survival (*p* = 0.001, B) compared to those whose tumors are ASS1-high in the untreated cohort.** (C) and (D) Kaplan–Meier survival curves showing that ASS1-low is associated reduced overall survival (p = 0.04, C), but not disease-free survival (*p* = 0.13, D) compared to those whose tumors are ASS1-high in the treated cohort.

The median follow-up time was 21.8 months (range: 4.2–236.3 months) for the overall untreated cohort and 41.2 months (range: 4.7–236.3 months) for patients who did not die from disease. At the time of last follow-up, 94 (69.6%) patients died of PDAC, 3 (2.2%) died of other causes, 7 (5.2%) patients were alive with disease and 31 (23.0%) were alive with no clinical or radiographic evidence of disease. The median DFS and OS was 4.8 ± 1.6 months and 14.6 ± 6.4 months respectively in ASS1-low group compared to15.3 ± 2.2 months (p = 0.001) and 26.5 ± 3.5 months (p = 0.005) respectively in ASS-high group (Fig 4A and 4B). By univariate analysis, ASS1 expression, tumor size (greater than 2.0 cm), margin status, lymph node status and AJCC stage correlated significantly with both DFS and OS (p<0.05, Table 2). By multivariate analysis, ASS1 expression [hazard ratio (HR): 0.45, 95% confidence interval (CI): 0.26–0.78) was an independent prognostic factor for DFS (p = 0.005), but not OS (p = 0.06, Table 3).

**Table 2:** Univariate Cox Regression Analysis of Disease-free and Overall Survival in Untreated Group.

Characteristics	No. of patients	Disease-free Survival		Overall Survival	
		HR (95% CI)	*p* value	HR (95% CI)	*p* value
**ASS1 expression**					
Low (ref)	16	1.00		1.00	
High	119	0.39 (0.22–0.68)	**0.001**	0.45 (0.25–0.80)	**0.007**
**Age (years)**					
≤ 60 (ref)	39	1.00		1.00	
60–70	56	1.06 (0.66–1.71)	0.81	1.30 (0.79–2.12)	0.3
≥ 70	40	1.39 (0.83–2.34)	0.21	1.64 (0.96–2.83)	0.07
**Gender**					
Female (ref)	58	1.00		1.00	
Male	77	1.19 (0.97–1.45)	0.09	1.13 (0.82–1.56)	0.46
**Tumor size**					
≤ 2cm (ref)	19	1.00		1.00	
> 2 cm	116	3.73 (1.71–8.13)	**0.001**	3.25 (1.56–6.81)	**0.002**
**Tumor differentiation**					
Well-Moderate (ref)	94	1.00		1.00	
Poor	41	0.91 (0.59–1.41)	0.12	0.89 (0.57–1.40)	0.62
**Margins**					
Negative (ref)	115	1.00		1.00	
Positive	20	1.78 (1.06–2.98)	**0.03**	1.87 (1.10–3.19)	**0.02**
**Lymph node status**					
Negative (ref)	33	1.00		1.00	
Positive	102	2.60 (1.53–4.42)	**<0.001**	2.02 (1.18–3.45)	**0.01**

Abbreviations: HR: hazard ratio; 95% CI: 95% confidence interval

**Table 3:** Multivariate Cox Regression Analysis of Disease-free and Overall Survival in Untreated Group.

Characteristics	No. of patients	Disease-free Survival		Overall Survival	
		HR (95% CI)	*p* value	HR (95% CI)	*p* value
**ASS1 expression**					
Low (ref)	16	1.00		1.00	
High	119	0.45 (0.26–0.79)	**0.005**	0.57 (0.32–1.03)	0.06
**Tumor size**					
≤ 2.0 cm (ref)	19	1.00		1.00	
> 2.0 cm	116	2.48 (1.11–5.55)	**0.03**	2.44 (1.12–5.28)	**0.02**
**Margins**					
Negative (ref)	115	1.00		1.00	
Positive	20	1.81 (1.07–3.07)	**0.03**	1.65 (0.96–2.82)	0.07
**Lymph node status**					
Negative (ref)	33	1.00		1.00	
Positive	102	2.26 (1.31–3.89)	**0.003**	1.58 (0.91–2.77)	0.11

Abbreviations: HR: hazard ratio; 95% CI: 95% confidence interval; ref: reference

### ASS1 expression correlated with overall survival in treated cohort

The median follow-up time was 33.0 months (range: 7.6–171.9 months) for the overall treated cohort and 107.1 months (range: 9.4–171.9 months) for patients who did not die from disease. At the time of last follow-up, 91 (74.6%) patients died of PDAC, 1 (0.8%) died of other causes, 1 (0.8%) patients were alive with disease and 29 (23.8%) were alive with no clinical or radiographic evidence of disease. The median OS was 16.5 ± 5.2 months in ASS1-low group compared to 35.3 ± 2.8 months in ASS-high group (p = 0.04, Fig 4C). The median DFS was 7.2 ± 2.0 months in ASS1-low group compared to 15.7 ± 3.7 months in ASS-high group (p = 0.13, Fig 4D). By univariate analysis, ASS1 expression, lymph node status and AJCC stage correlated significantly with OS (p<0.05, Table 4). By multivariate analysis, both ASS1 expression (HR: 0.56, 95% CI: 0.32–0.97, p = 0.04) and lymph node metastasis (HR: 1.57, 95% CI: 1.004–2.47, p = 0.048) were independent prognostic factors for OS (Table 5)

**Table 4:** Univariate Cox Regression Analysis of Overall Survival in Treated Group.

Characteristics	No. of patients	Overall Survival	
		HR (95% CI)	*p* value
**ASS1 expression**			
Low (ref)	18	1.00	
High	104	0.56 (0.32–0.98)	0.04
**Age (years)**			
≤ 60 (ref)	50	1.00	
60–70	44	0.85 (0.53–1.34)	0.48
≥ 70	28	0.76 (0.44–1.30)	0.32
**Neoadjuvant regimens**			
Fluoropyrimidine-Rad	18	1.00	
Gem-Rad	39	1.04 (0.53–2.07)	0.90
Gem-based with GemRad	45	1.48 (0.77–2.85)	0.24
Gem-based-FPRad	15	1.25 (0.56–2.78)	0.59
Chemo alone	5	1.65 (0.53–5.14)	0.39
**Gender**			
Female (ref)	49	1.00	
Male	73	1.10 (0.90–1.36)	0.35
**Tumor size**			
≤ 2cm (ref)	30	1.00	
> 2 cm	92	1.05 (0.64–1.72)	0.85
**Tumor differentiation**			
Well-Moderate (ref)	78	1.00	
Poor	44	1.24 (0.81–1.90)	0.31
**Margins**			
Negative (ref)	110	1.00	
Positive	12	1.61 (0.83–3.11)	0.16
**Lymph node status**			
Negative (ref)	41	1.00	
Positive	81	1.57 (1.00–2.45)	0.05

Abbreviations: HR: hazard ratio; 95% CI: 95% confidence interval

**Table 5:** Multivariate Cox Regression Analysis of Overall Survival in Treated Group.

Characteristics	No. of patients	Overall Survival	
		HR (95% CI)	*p* value
**ASS1 expression**			
Low (ref)	18	1.00	
High	104	0.56 (0.32–0.97)	0.04
**Tumor differentiation**			
Well-Moderate (ref)	78	1.00	
Poor	44	1.17 (0.74–1.84)	0.51
**Margins**			
Negative (ref)	110	1.00	
Positive	12	1.38 (0.70–2.71)	0.36
**Lymph node status**			
Negative (ref)	41	1.00	
Positive	81	1.57 (1.00–2.47)	0.048

Abbreviations: HR: hazard ratio; 95% CI: 95% confidence interval

## Discussion

Pathologic factors that are known to confer a negative prognostic impact in patients with PDAC include high pathologic primary tumor (pT) stage [16–18], poor tumor differentiation [16, 19–21], positive lymph node status [16, 18, 20–24] etc. Our study identified ASS1 as a new prognostic factor in patients with PDAC in two large independent cohorts of patients: 135 patients who underwent upfront PD without neoadjuvant therapy (untreated cohort) and 122 patients received pre-operative neoadjuvant therapy and PD (treated cohort). We showed that low level of ASS1 expression (ASS1-low) is associated with higher recurrence after PD and is an independent negative prognostic factor for survival in both untreated cohort and treated cohort. Our data indicated that ASS1 might represent a valuable marker for early prognostic evaluation in patients with PDAC. ASS1 expression can be evaluated on surgical biopsy specimens or cell blocks for fine needle aspiration of pancreatic tumor. A low ASS1 combined score (less than 1.5) will help identify patients with poor prognosis, and patients more likely with better response to arginine deprivation therapy in the early course of the disease. Similar to what we reported here, a deficient expression of ASS1 has been previously described to be associated with clinical aggressiveness in myxofibrosarcomas, bladder cancer and nasopharyngeal carcinoma [11–13]. The negative prognostic impact associated with deficient ASS1 might be attributed to the newly identified tumor suppressor function of ASS1 besides its functions in arginine metabolism. Huang et al showed that expression of *Ass1* gene inhibits tumoral angiogenesis, tumor growth, cell migration and invasion in myxofibrosarcomas, while knockdown of *Ass1* gene confers tumor proliferative and metastatic capabilities [11].

ASS1 is present ubiquitously in mammals, making arginine a nonessential amino acid. However, expression of ASS1 could be quite different in various tissues, depending on the need of tissues for arginine. Similarly, ASS1 expression is greatly variable in human malignant tumors. Majority of the lung and colon carcinomas show ASS1 expression, while melanoma, hepatocellular carcinoma and prostate carcinomas are frequently ASS1-deficient [7]. ASS1 deficiency in the latter may make these tumors sensitive to external arginine depletion. Several regulatory mechanisms including hormones, nutrients, pro-inflammatory cytokines have been described to be involved in regulation of *Ass1* gene expression [25]. Recently, Huang et al. and Lan et al. for the first time demonstrated at the molecular level that loss of ASS1 protein expression is strongly linked to *Ass1* promotor hypermethylation [11, 13]. In our study, we found that low expression of ASS1 in 12% and 15% of the untreated and treated PDAC samples respectively. Whether the low expression of ASS1 in PDAC is attributed to *Ass1* promotor hypermethylation needs to be examined in future studies.

In our study, ASS1 expression is significantly lower in patients treated with pre-operative neoadjuvant therapy (treated cohort) than that in untreated cohort, implicating a role of neoadjuvant therapy in regulation of ASS1 expression. This might occur through a direct regulation of ASS1 expression by neoadjuvant therapy at transcriptional, epigenetic e.g. hypermethylation of *Ass1* promotor or translational level, or through altered pro-inflammatory cytokines induced by neoadjuvant therapy. Alternatively, neoadjuvant therapy might apply a selection pressure on tumor cells; with those express low ASS1 (more aggressive phenotype and more resistant to therapy) survive.

Our study showed that 12% of PDAC in untreated cohort and 15% of PDAC in treated cohort has low expression of ASS1 (ASS1-low). These patients may benefit from arginine depletion therapy using PEG-ADI either alone or in combination with conventional chemoradiation therapies. In addition, patients whose tumors have reduced ASS1 expression after neoadjuvant therapy may also be susceptible to arginine deprivation. Consistent with this notion, previous study has shown that five of seven pancreatic cancer cell lines lacked ASS1 expression and that arginine deprivation by treatment with PEG-ADI specifically inhibited the growth of pancreatic cancer cell lines that lack ASS1 expression both *in vitro* and *in vivo* [26]. However, our data indicated that majority of the PDAC patients might not benefit from arginine deprivation treatment as their tumor have sufficient ASS1 protein for endogenous arginine production. Interestingly, Daylami et al. showed that PEG-ADI synergistically increases the cytotoxicity of gemcitabine in human pancreatic cancer cell lines both *in vitro* and *in vivo* [5, 10]. The underlying mechanisms are unclear, and the authors proposed that arginine deprivation induces cellular changes that re-program cells allowing sensitization to traditional chemotherapy [5, 27]. This hypothesis, however, is only meaningful when majority of the tumor cells expressing deficient/low ASS1.

In summary, our study showed that a small percentage of PDAC has low expression of ASS1 and that ASS1 expression is reduced in patients who received neoadjuvant therapies. Low level ASS1 expression is associated with higher frequency of recurrence after surgery and is associated with shorter survival in both untreated and treated PDAC patients. Our findings provide supporting evidence for future clinical trial using arginine deprivation agents either alone or in combination with gemcitabine and other conventional chemotherapy agents in treating pancreatic cancer.
